# Maternal and perinatal outcomes in vasculitis: a 15-year study at a Portuguese tertiary multidisciplinary centre

**DOI:** 10.1093/rap/rkag002

**Published:** 2026-01-07

**Authors:** Ana Rita Lopes, Susana Capela, Ana Rita Cruz-Machado, Ana T. Chícharo, Sofia C Barreira, Patrícia Martins, Maria João Saavedra, Maria Pulido Valente, Luísa Pinto, Cristina Ponte

**Affiliations:** Rheumatology Department, ULS Santa Maria, Lisbon Academic Medical Centre, Lisbon, Portugal; Faculdade de Medicina, Universidade de Lisboa, Lisbon Academic Medical Centre, Lisbon, Portugal; Rheumatology Department, ULS Santa Maria, Lisbon Academic Medical Centre, Lisbon, Portugal; Faculdade de Medicina, Universidade de Lisboa, Lisbon Academic Medical Centre, Lisbon, Portugal; Rheumatology Department, ULS Santa Maria, Lisbon Academic Medical Centre, Lisbon, Portugal; Faculdade de Medicina, Universidade de Lisboa, Lisbon Academic Medical Centre, Lisbon, Portugal; Rheumatology Department, ULS Santa Maria, Lisbon Academic Medical Centre, Lisbon, Portugal; Rheumatology Department, ULS Algarve, Faro, Portugal; Rheumatology Department, ULS Santa Maria, Lisbon Academic Medical Centre, Lisbon, Portugal; Faculdade de Medicina, Universidade de Lisboa, Lisbon Academic Medical Centre, Lisbon, Portugal; Rheumatology Department, ULS Santa Maria, Lisbon Academic Medical Centre, Lisbon, Portugal; Rheumatology Department, ULS Santa Maria, Lisbon Academic Medical Centre, Lisbon, Portugal; Faculdade de Medicina, Universidade de Lisboa, Lisbon Academic Medical Centre, Lisbon, Portugal; Obstetrics, Gynecology, and Reproductive Medicine Department, ULS Santa Maria, Lisbon Academic Medical Centre, Lisbon, Portugal; Faculdade de Medicina, Universidade de Lisboa, Lisbon Academic Medical Centre, Lisbon, Portugal; Obstetrics, Gynecology, and Reproductive Medicine Department, ULS Santa Maria, Lisbon Academic Medical Centre, Lisbon, Portugal; Rheumatology Department, ULS Santa Maria, Lisbon Academic Medical Centre, Lisbon, Portugal; Faculdade de Medicina, Universidade de Lisboa, Lisbon Academic Medical Centre, Lisbon, Portugal

**Keywords:** vasculitis, pregnancy outcomes, disease relapse, remission, maternal health, perinatal care

## Abstract

**Objectives:**

To evaluate maternal and perinatal outcomes in women with vasculitis and identify risk factors for adverse pregnancy outcomes (APOs).

**Methods:**

This retrospective study included pregnant women with systemic vasculitis followed between 2009 and 2024 at a multidisciplinary tertiary centre. Clinical data on patient characteristics, disease activity (remission or relapse), treatment and pregnancy outcomes were collected. APOs comprised miscarriage, stillbirth, preterm birth, foetal growth restriction (FGR), small for gestational age (SGA) and pre-eclampsia. Associations between clinical variables and APOs were assessed using univariate analysis and multivariate binary logistic regression.

**Results:**

A total of 39 pregnancies occurred in 28 women with vasculitis. Most pregnancies (92.3%) occurred during clinical remission. The most frequent vasculitis subtypes were Behçet’s disease (*n* = 22), polyarteritis nodosa (*n* = 5) and Takayasu arteritis (*n* = 5). Only 17.9% of pregnancies were preceded by formal preconception counselling. Disease relapses were observed in 14 pregnancies (35.9%) and 5 postpartum periods (12.8%). APOs were reported in 13 pregnancies (33.3%), including 3 miscarriages (7.7%), 6 preterm births (15.4%), 6 SGA (15.4%), 2 FGR (5.1%) and 1 pre-eclampsia (2.6%). Prednisolone use and relapses during pregnancy were independently associated with APOs [odds ratio (OR) 26.10, *P* = 0.024; OR 8.90, *P* = 0.019). No associations were found for maternal age, cumulative prednisone dose or use of immunosuppressants. No maternal deaths or severe neonatal complications occurred.

**Conclusion:**

Pregnancy in women with vasculitis is often successful in the context of clinical remission at conception. Our findings are exploratory but suggest that prednisolone use and disease relapse may increase the risk of APOs, highlighting the importance of preconception counselling and multidisciplinary care throughout pregnancy.

Key messages

- Pregnancy can be successful in vasculitis when planned in remission and under multidisciplinary specialist care.
- Prednisolone exposure and disease relapse are associated with adverse outcomes, although these findings remain exploratory.
- Low preconception counselling rates highlight a modifiable gap and actionable opportunity to improve maternal and perinatal outcomes.

## Introduction

Vasculitides are heterogeneous autoimmune disorders characterised by blood vessel inflammation, potentially causing multi-organ damage [1–3]. Takayasu arteritis (TAK), polyarteritis nodosa (PAN) and Behçet’s disease (BD) are commonly diagnosed in women of reproductive age, although rarer forms may also occur [1, 2, 4].

Despite advances in immunosuppressive therapy, pregnancies in women with vasculitis remain high risk [2]. Favourable outcomes are more likely when disease is in remission at conception and remains controlled throughout gestation [1, 5]. However, even with these strategies, maternal and foetal complications remain common. Balancing maternal disease control with foetal safety is increasingly feasible with pregnancy-compatible medications. Nevertheless, real-world data on pregnancy outcomes in vasculitis remain scarce. In particular, evidence on how disease activity and vasculitis subtype influence maternal and perinatal outcomes is limited and largely based on small studies [2, 6–8].

This study aims to describe maternal and perinatal outcomes over a 15-year period at a Portuguese tertiary multidisciplinary centre and explore clinical factors associated with adverse pregnancy outcomes (APOs), contributing to a better understanding and care of these pregnancies.

## Methods

### Study design and population

A retrospective observational study was conducted involving pregnant women with systemic vasculitis followed at a multidisciplinary rheumatology–obstetrics clinic within a tertiary academic centre between 2009 and 2024. The clinic is jointly managed by rheumatologists and maternal–foetal medicine specialists, with expertise in inflammatory rheumatic diseases and high-risk pregnancies.

This study was approved by the Ethics Committee of the Lisbon Academic Medical Centre (reference 228/24). All procedures were performed in accordance with the ethical standards laid down in the 1964 Declaration of Helsinki and its later amendments.

### Data collection and definitions

Data were extracted from medical records following institutional ethics approval (reference 228/24). Collected variables included demographics, vasculitis subtype, disease duration and disease activity and management at conception and throughout pregnancy. Prednisolone exposure was assessed as daily dose at conception and cumulative dose throughout pregnancy. Disease activity was defined as remission or relapse based on clinical symptoms, laboratory markers, imaging and biopsy findings. Remission was defined as the absence of symptoms, normal inflammatory markers and stable organ function. Relapse was defined as new or worsening vasculitis-related manifestations during pregnancy or within 6 weeks postpartum.

### Outcomes

Maternal and perinatal outcomes included miscarriage (<22 weeks) and stillbirth (≥22 weeks), according to the definitions adopted in Portugal and consistent with World Health Organization/Eurostat criteria [9–11], as well as pre-eclampsia [new-onset hypertension (systolic blood pressure ≥140 mmHg or diastolic blood pressure ≥90 mmHg) after 20 weeks of gestation or postpartum, with proteinuria (>300 mg/24 h) or maternal organ dysfunction] and eclampsia (generalised seizures in the context of pre-eclampsia). Gestational diabetes mellitus was also reviewed. Foetal and neonatal outcomes included preterm birth (<37 weeks), foetal growth restriction (FGR, estimated foetal weight <3rd percentile or <10th percentile with abnormal Doppler findings), SGA (birth weight <10th percentile), congenital anomalies (structural abnormalities identified at birth) and neonatal infections within 28 days of life [12–14].

### Statistical analysis

Descriptive statistics summarised clinical and pregnancy data. Categorical variables are presented as frequencies and percentages and continuous variables as means (s.d.s) or medians [interquartile ranges (IQRs)]. Distribution normality was assessed using the Shapiro–Wilk test, skewness and kurtosis. Associations between categorical variables were tested using the chi-squared test or Fisher’s exact test. Continuous variables were compared using the Student’s t-test or Mann–Whitney U test, as appropriate. To explore independent predictors of APOs, defined as a composite binary outcome, we performed a multivariate binary logistic regression analysis. Predictor variables were selected based on clinical relevance and/or statistical significance in univariate analyses. Because some women contributed more than one pregnancy, intra-individual clustering was considered; however, robust variance estimation was not applied, owing to the limited number of observations per subject. Analyses were therefore interpreted descriptively and exploratorily. Statistical significance was set at *P* < 0.05 (two-tailed). Odds ratios (ORs) with 95% CIs were calculated for significant associations. Analyses were performed using SPSS Statistics version 30.0 (IBM, Armonk, NY, USA).

## Results

### Patient characteristics

Twenty-eight women with systemic vasculitis were included. The most prevalent diagnoses were BD [*n* = 14 (50.0%)], PAN [*n* = 4 (14.3%)] and TAK [*n* = 4 (14.3%)]. Other subtypes included cryoglobulinaemic vasculitis, IgA vasculitis, ANCA-PR3 cutaneous vasculitis, relapsing polychondritis, eosinophilic granulomatosis with polyangiitis (EGPA) and cryoglobulinaemic vasculitis associated with Sjögren’s syndrome [*n* = 1 (3.6%) each].

Thirty-nine pregnancies were analysed. The mean maternal age at conception was 32.9 years (s.d. 5.6) and the mean disease duration was 8.7 years (s.d. 6.4) years. Clinical and obstetric characteristics are summarised in Supplementary Table S1 and Supplementary Fig. S1.

### Disease status and management at conception

Only one woman, presenting with PAN, was newly diagnosed during pregnancy; all other women had a prior diagnosis. Only seven pregnancies (17.9%) had formal preconception counselling at our joint clinic. Most pregnancies [*n* = 36 (92.3%)] occurred during clinical remission. Three (7.7%) began with active disease (two PAN, one IgA vasculitis). Maintenance therapy at conception included glucocorticoids [*n* = 18 (46.2%)], conventional synthetic DMARDs [*n* = 8 (20.5%)] and biologic DMARDs [*n* = 2 (5.1%)]. Anti-phospholipid antibodies were detected in two patients (one TAK, one BD), with no APOs reported.

### Treatment during conception and pregnancy

Prednisolone was prescribed in 27 pregnancies (69.2%), with a median cumulative dose during pregnancy of 664 mg (IQR 0–1310). Eighteen pregnancies (46.2%) were already on prednisolone at the time of conception, with a median daily dose of 2.5 mg/day (IQR 0–5). Four pregnancies were exposed to >7.5 mg/day at conception: two TAK (10 and 15 mg/day), one PAN (30 mg/day) and one ANCA-associated cutaneous vasculitis (10 mg/day). During gestation, seven pregnancies required doses >7.5 mg/day (three PAN, three TAK and one ANCA-associated cutaneous vasculitis).

Conventional DMARDs were used in 11 pregnancies (28.2%), including hydroxychloroquine (*n* = 2) and azathioprine (*n* = 6), and both agents in three cases. Colchicine was administered in four pregnancies with BD. No pregnancies were exposed to dapsone, ciclosporin or tacrolimus. Biologic DMARDs were continued in two TAK pregnancies with severe vascular involvement. One patient with TAK in remission at conception was receiving subcutaneous tocilizumab (162 mg/week). During pregnancy, a thoracic aortic aneurysm progressed from 47 to 56 mm in diameter and caesarean delivery was performed five days after the last tocilizumab dose at 32 weeks due to the risk of rupture. Another TAK patient with multisegmented aortic disease and bilateral carotid stenosis became pregnant without preconception counselling and had active disease. Tocilizumab (162 mg/week) was maintained until week 30 and elective caesarean was performed at week 37. Both neonates were healthy at the 6-month follow-up.

### Disease relapses and postpartum course

Relapses occurred during 14 pregnancies (35.9%) and 5 postpartum periods (12.8%). Most were mild, involving mucocutaneous or articular symptoms, and were managed successfully with glucocorticoid or colchicine adjustment. No major organ-threatening relapses or maternal deaths occurred.

### Pregnancy and neonatal outcomes

APOs were observed in 13/39 pregnancies (33.3%): 3 first-trimester miscarriages (7.7%), 6 preterm births (15.4%), 6 SGA newborns (15.4%) and 2 FGR (5.1%). No cases of gestational diabetes mellitus were observed. The median gestational age at delivery was 38.0 weeks (IQR 38.0 (2.0)). Most preterm births were late preterm (32–36 weeks). The mean birth weight was 2930 g (s.d. 518; range 1725–4010), with low birth weight (<2500 g) in six newborns (15.4%). One case of pre-eclampsia occurred in a patient with PAN, chronic hypertension and nulliparity. She was on methyldopa but at 37 weeks developed worsening hypertension, visual disturbances and proteinuria, prompting induction of labour.

No stillbirths, medically indicated terminations, congenital anomalies or serious neonatal infections were reported. Caesarean delivery was performed in 12 cases (33.3%), mostly for obstetric indications. Individual pregnancy outcomes are shown in Supplementary Table S1.

Prednisolone exposure during pregnancy was associated with a significantly higher rate of APOs than no exposure [12/27 (44.4%) *vs* 1/12 (8.3%), *P* = 0.034]. In prednisolone-exposed pregnancies, no differences were observed in the occurrence of APOs between those treated with >7.5 mg/day and those treated with ≤7.5 mg/day at conception [2/4 (50.0%) *vs* 6/14 (42.9%) APOs, *P* = 1.000) and throughout gestation [10/20 (50.0%) *vs* 2/7 (28.6%) APOs, *P* = 0.408]. APOs were also more frequent in patients with disease relapse during pregnancy than among those who remained stable [8/14 (57.1%) *vs* 5/25 (20.0%), *P* = 0.033]. No significant differences were observed in cumulative prednisolone dose [median 1015.0 mg (IQR 1188.0) *vs* 579.0 mg (IQR 1157.0), *P *= 0.160), use of immunosuppressants [4/12 (33.3%) *vs* 9/27 (33.3%), *P* = 1.000) or maternal age [median 35.0 years (IQR 9.0) *vs* 34.0 years (IQR 7.0), *P* = 0.303].

In multivariate analysis adjusted for maternal age, cumulative prednisone dose and immunosuppressants, both prednisolone use [OR 26.10 (95% CI 1.54, 440.83), *P* = 0.024] and disease relapse during pregnancy [OR 8.90 (95% CI 1.44, 56.83), *P* = 0.019] remained independently associated with APOs.

### Outcomes by vasculitis subtypes

Among the 22 pregnancies in patients with BD, 6 (27.3%) had APOs: 1 miscarriage (4.5%), 3 SGA newborns (13.7%) and 4 preterm births (18.2%); disease relapses occurred in 8 pregnancies (36.4%) and in four postpartum periods (19.0%).

In the PAN group (*n* = 5), 2 pregnancies (40%) had APOs: 1 FGR (20.0%) and 1 with pre-eclampsia and SGA (20.0%); disease relapses occurred in 3 cases (60.0%). For TAK (*n* = 5), 2 pregnancies (40.0%) had APOs: 1 miscarriage (20.0%) and 1 preterm birth (25%); only one disease relapse (20.0%) occurred.

In other subtypes, the patient with ANCA-PR3 cutaneous vasculitis experienced both SGA and disease relapse; the patient with relapsing polychondritis experienced multiple complications, including SGA, FGR, preterm birth and disease relapse during both pregnancy and postpartum, and the patient with EGPA experienced a first-trimester miscarriage despite clinical remission, suggesting a non-disease-related cause. Table 1 summarizes outcomes by vasculitis subtype.

**Table:** **Table 1** Maternal and perinatal outcomes according to vasculitis subtype.

Main diagnosis	Pregnancies, *n* (%)	Miscarriages, *n* (%)	SGA, *n* (%)	FGR, *n* (%)	Preterm birth, *n* (%)	Pre-eclampsia, *n* (%)	Pregnancy relapses, *n* (%)	Postpartum relapses, *n* (%)	Pregnancies with APOs, *n* (%)
Behçet’s disease	22 (56.4)	1 (4.5)	3 (13.7)	0	4 (18.2)	0	8 (36.4)	4 (19.0)	6 (27.3)
Polyarteritis nodosa	5 (12.8)	0	1 (20.0)	1 (20.0)	0	1 (20.0)	3 (60.0)	0	2 (40.0)
Takayasu arteritis	5 (12.8)	1 (20.0)	0	0	1 (20.0)	0	1 (20.0)	0	2 (40.0)
IgA vasculitis	1 (2.6)	0	0	0	0	0	0	0	0
ANCA-PR3 cutaneous vasculitis	1 (2.6)	0	1	0	0	0	1	0	1
Relapsing polychondritis	1 (2.6)	0	1	1	1	0	1	1	1
Cryoglobulinaemic vasculitis	2 (5.1)	0	0	0	0	0	0	0	0
Cryoglobulinaemic vasculitis associated with SS	1 (2.6)	0	0	0	0	0	0	0	0
Eosinophilic granulomatosis with polyangiitis	1 (2.6)	1	0	0	0	0	0	0	1
Total	39 (100.0)	3 (7.7)	6 (15.4)	2 (5.1)	6 (15.4)	1 (2.6)	14 (35.9)	5 (12.8)	13 (33.3)

## Discussion

This study demonstrates that pregnancy in women with vasculitis is frequently associated with favourable outcomes, particularly when conception occurs during clinical remission, in line with previous evidence [1, 3, 4, 6]. Although our study was not powered to show a formal association, this remains an important clinical principle. Nonetheless, relapses and APOs were not uncommon, affecting more than one-third of pregnancies, highlighting the need for close surveillance and multidisciplinary care throughout gestation.

In our cohort, prednisolone use and disease relapse during pregnancy were independently associated with an increased risk of APOs, reinforcing the importance of achieving remission before conception and maintaining tight disease control during gestation. Glucocorticoids remain essential for managing vasculitis in pregnancy, and their use was more frequent in pregnancies with APOs. However, the small sample size and wide CIs preclude determining an independent glucocorticoid effect, so these findings should be interpreted with caution.

Glucocorticoids were more frequently prescribed to women with active or more severe disease, making confounding by indication the most plausible explanation. The absence of a dose–response relationship and the lack of association with cumulative doses support this interpretation. Prior studies have similarly reported inconsistent findings regarding glucocorticoid-related risks [2, 15].

The overall relapse rate of 35.9% aligns with previous reports (20–40%), varying by vasculitis subtype [3, 8]. BD and PAN showed higher rates of relapse and APOs, whereas TAK was generally more stable in our cohort, despite larger studies reporting increased risks of pre-eclampsia, miscarriage, FGR and preterm birth in TAK [16]. Two TAK patients with aortic involvement required continued biologic therapy. Pregnancy-related increases in blood volume and cardiac workload may worsen pre-existing vascular lesions, as illustrated by a thoracic aortic aneurysm progression requiring early delivery [8, 16]. Notably, one patient unexpectedly improved during gestation (resolution of carotidynia and presyncope), but relapsed postpartum [17].

Another key finding is the low rate of preconception counselling (17.9%) despite specialised multidisciplinary management. This likely reflects unplanned pregnancies and delayed referral. Improving access and referral pathways for counselling should be considered one of the most actionable messages from our study, with clear potential to optimise outcomes.

Most patients received low-dose glucocorticoids, either as monotherapy or combined with azathioprine or hydroxychloroquine [15]. In two TAK cases, tocilizumab was continued throughout pregnancy due to life-threatening disease, without apparent adverse neonatal effects [17]. Current recommendations emphasise controlling maternal disease activity, including continuation of biologics when necessary. While discontinuation in the third trimester is often suggested to minimise neonatal immunosuppression, exceptions may apply in severe or refractory disease [1, 2, 15, 18, 19].

These findings highlight the importance of individualised preconception counselling and striving for remission before conception [2]. Even with remission at conception, relapses may still occur during gestation or postpartum, supporting the need for close, multidisciplinary monitoring. Postpartum surveillance is particularly important given the risk of disease reactivation in the early puerperium. Prior pregnancy outcomes should not be assumed to be predictive of future courses, and each vasculitis subtype presents distinct pregnancy risks that warrant tailored assessment and follow-up [2, 3, 8].

A key strength of this study is the granularity of real-world data collected over 15 years at a multidisciplinary academic centre, providing longitudinal insights into a rare patient population. Strict clinical definitions for remission and relapse add consistency and internal validity. Validated disease activity scores (e.g. Birmingham Vasculitis Activity Score, Indian Takayasu Arteritis Score or Behçet’s Disease Current Activity Form) were not systematically available in the historical record; disease status was therefore classified as remission or relapse based on clinical judgement, laboratory markers and imaging, which limits comparability across studies. Including rarer subtypes, such as EGPA and relapsing polychondritis, broadens the relevance of our findings. The retrospective design entails potential reporting bias and incomplete data capture, rarity and heterogeneity limited statistical power for subtype-specific analyses and the small sample yielded wide CIs for risk estimates. Consequently, our analyses should be regarded as primarily descriptive, with exploratory associations rather than definitive evidence. Finally, the predominance of BD reflects national epidemiology [20], supporting generalisability to similar populations while cautioning against extrapolation to settings with different disease distributions.

## Conclusion

Pregnancy in women with vasculitis often has favourable outcomes when the disease is well controlled under specialist care. However, relapse and APOs remain common, reinforcing the need for individualised preconception planning, close monitoring during pregnancy and postpartum and coordinated multidisciplinary care. Our experience supports the role of joint rheumatology–obstetrics clinics to optimise pregnancy outcomes in this population.

## Supplementary Material

rkag002_Supplementary_Data

## Data Availability

The data underlying this article will be shared on reasonable request to the corresponding author.

## References

[1] Sims CA , Bermas BL, Clowse MEB. Vasculitis and pregnancy: disease state and management. Rheum Dis Clin North Am 2023;49:679–94. 37331740 10.1016/j.rdc.2023.03.009

[2] Sims C , Clowse MEB. A comprehensive guide for managing the reproductive health of patients with vasculitis. Nat Rev Rheumatol 2022;18:711–23. 36192559 10.1038/s41584-022-00842-z PMC9529165

[3] Beça S , Alba MA, Hernández-Rodríguez J et al Maternal and fetal outcomes of pregnancy in women with primary systemic vasculitis: a single-centre cohort study of 20 patients and 30 pregnancies. Semin Arthritis Rheum 2024;66:152412. 38387195 10.1016/j.semarthrit.2024.152412

[4] Nguyen V , Wuebbolt D, Pagnoux C, D’Souza R. Pregnancy outcomes in women with primary systemic vasculitis: a retrospective study. J Matern Fetal Neonatal Med 2021;34:2771–7. 31571516 10.1080/14767058.2019.1671329

[5] Mendel A , Vinet É. Connecting the docs in vasculitis pregnancies. J Rheumatol 2024;51:949–52. 39218452 10.3899/jrheum.2024-0733

[6] Sangle SR , Vounotrypidis P, Briley A et al Pregnancy outcome in patients with systemic vasculitis: a single-centre matched case-control study. Rheumatology (Oxford) 2015;54:1582–6. 25832613 10.1093/rheumatology/kev018

[7] Sims CA , Eudy AM, Larson K, et al Reproductive outcomes for women with vasculitis. J Rheumatol 2024;51:1003–8. 38825354 10.3899/jrheum.2023-1246 PMC13173927

[8] Gatto M , Iaccarino L, Canova M et al Pregnancy and vasculitis: a systematic review of the literature. Autoimmun Rev 2012;11:A447–59. 22155197 10.1016/j.autrev.2011.11.019

[9] Euro-Peristat Project. European perinatal health report. Core indicators of the health and care of pregnant women and babies in Europe in 2015. Paris: Inserm, 2018. http://www.europeristat.com

[10] World Health Organization. International statistical classification of diseases and related health problems, 10th revision. 5th ed. Geneva: World Health Organization, 2016.

[11] Instituto Nacional de Estatística, Direção-Geral da Saúde. Nados-vivos e óbitos fetais: Notas metodológicas e definições. Lisbon: INE/DGS, 2022. https://www.ine.pt

[12] American College of Obstetricians and Gynecologists’ Committee on Practice Bulletins—Obstetrics. ACOG practice bulletin No. 203: chronic hypertension in pregnancy. Obstet Gynecol 2019;133:e26–50. 30575676 10.1097/AOG.0000000000003020

[13] Lees CC , Stampalija T, Baschat A et al ISUOG practice guidelines: diagnosis and management of small-for-gestational-age fetus and fetal growth restriction. Ultrasound Obstet Gynecol 2020;56:298–312. 32738107 10.1002/uog.22134

[14] American College of Obstetricians and Gynecologists’ Committee on Practice Bulletins—Obstetrics. Practice bulletin no. 171: management of preterm labor. Obstet Gynecol 2016;128:e155–64. 27661654 10.1097/AOG.0000000000001711

[15] Sammaritano LR , Bermas BL, Chakravarty EE et al 2020 American College of Rheumatology guideline for the management of reproductive health in rheumatic and musculoskeletal diseases. Arthritis Rheumatol 2020;72:529–56. 32090480 10.1002/art.41191

[16] Partalidou S , Mamopoulos A, Dimopoulou D, Dimitroulas T. Pregnancy outcomes in Takayasu arteritis patients: a systematic review and meta-analysis. Sci Rep 2023;13:546. 36631504 10.1038/s41598-023-27379-9 PMC9834209

[17] Cruz-Machado AR , Andrade Silva L, Barreira SC et al Tocilizumab throughout pregnancy in two patients with severe Takayasu’s arteritis. Acta Reumatol Port 2021;46:193–5. 34243184

[18] Rüegg L , Pluma A, Hamroun S et al EULAR recommendations for use of antirheumatic drugs in reproduction, pregnancy, and lactation: 2024 update. Ann Rheum Dis 2025;84:910–26. 40287311 10.1016/j.ard.2025.02.023

[19] Russell MD , Dey M, Flint J et al British Society for Rheumatology guideline on prescribing drugs in pregnancy and breastfeeding: immunomodulatory anti-rheumatic drugs and corticosteroids. Rheumatology (Oxford) 2023;62:e48–88. 36318966 10.1093/rheumatology/keac551 PMC10070073

[20] Ponte C , Khmelinskii N, Teixeira V et al Reuma.pt/vasculitis—the Portuguese vasculitis registry. Orphanet J Rare Dis 2020;15:110. 32370776 10.1186/s13023-020-01381-0 PMC7201571

