# Supplementary material for: Maternal and perinatal outcomes in vasculitis: a 15-year study at a Portuguese tertiary multidisciplinary centre
Source: Rheumatol Adv Pract. 2026 Jan 7;10(1):rkag002. doi: 10.1093/rap/rkag002 (PMC12867576; doi:10.1093/rap/rkag002)
Supplement: rkag002_Supplementary_Data [file rkag002_supplementary_data.zip › rheumap-2025-212-18-23.pdf]

Supplementary Table S1. Clinical, Therapeutic and Pregnancy Data for 39 Pregnancies in Women with Systemic Vasculitis.

Patient	Diagnosis	Clinical Manifestations	Pregnancy Number	Maternal Age at Conception (years)	Disease Duration at conception (years)	Treatment		APOs	Delivery			Disease Activity/Relapses		
						At Conception	During pregnancy		Gestational age (weeks)	Delivery mode	Birth weight (g)	At Conception	During Pregnancy	During Postpartum
**1**	Behçet's Disease	Mucocutaneous: oral, genital, and perianal ulcers. Articular: arthritis. Cutaneous: folliculitis.	1	39	11	PDN≤7.5mg /day	PDN≤7.5mg /day	No	37+5	Eutocic	3000	No	No	Yes (oral ulcers)
			2	40	12	PDN≤7.5mg /day	PDN≤7.5mg /day	Yes (miscarriage at 8 WG)	*N/A*	*N/A*	*N/A*	No	No	*N/A*
			3	41	13	PDN≤7.5mg /day	PDN≤7.5mg /day	Yes (SGA)	37	Eutocic	2450	No	No	No
			4	43	14	PDN≤7.5mg /day; colchicine	PDN≤7.5mg /day	No	37+6	Eutocic	3095	No	Yes (oral ulcers)	No
**2**	Behçet's Disease	Mucocutaneous: oral and genital ulcers. Cutaneous: folliculitis. Vascular: thrombophlebitis.	1	33	6	None	PDN≤7.5mg /day	Yes (preterm birth; SGA)	32+4	Caesarean	1725	No	Yes (oral ulcers, folliculitis)	No
**3**	Behçet's Disease	Mucocutaneous: oral and genital ulcers. Cutaneous: folliculitis.	1	28	10	PDN≤7.5mg /day	PDN≤7.5mg /day	No	38	Dystocic	2730	No	No	No
**4**	Behçet's Disease	Mucocutaneous: oral and vaginal ulcers. Articular: arthralgias. Cutaneous: folliculitis.	1	36	6	None	PDN≤7.5mg /day	No	38	Eutocic	3610	No	No	No
			2	39	8	None	None	No	39+4	Eutocic	3185	No	No	No
**5**	Behçet's Disease	Mucocutaneous: oral and vaginal ulcers. Articular: arthralgias. Cutaneous: folliculitis.	1	31	9	None	None	No	39	Eutocic	2925	No	No	No
			2	34	12	None	None	No	38+4	Eutocic	999	No	No	No
**6**	Behçet's Disease	Mucocutaneous: oral and vaginal ulcers. Articular: arthritis. Gastrointestinal: diarrhea.	1	38	5	None	None	Yes (preterm birth)	35+5	Eutocic	2770	No	Yes (oral ulcers)	No

**7**	Behçet's Disease	Mucocutaneous: oral and vaginal ulcers. Cutaneous: erythema nodosum. Articular: arthralgias. Constitutional: fatigue.	1	35	20	PDN≤7.5mg /day	PDN≤7.5mg /day; colchicine	Yes (preterm birth; SGA)	35+2	Eutocic	2125	No	Yes (oral ulcers, erythema nodosum and fatigue)	Yes (oral ulcers, erythema nodosum)
			2	37	23	None	PDN≤7.5mg /day; colchicine	Yes (preterm birth)	35	Eutocic	2260	No	Yes (oral ulcers, fatigue)	Yes (oral ulcers)
**8**	Behçet's Disease	Mucocutaneous: oral and vaginal ulcers. Articular: arthralgias. Constitutional: fever.	1	36	3	None	None	No	40+3	Eutocic	3570	No	Yes (oral and vaginal ulcers)	No
**9**	Behçet's Disease	Mucocutaneous: oral ulcers. Articular: arthralgias.	1	36	12	None	None	No	39+4	Caesarean	3025	No	No	No
**10**	Behçet's Disease	Mucocutaneous: oral and vaginal ulcers. Neurologic: meningo-encephalitis.	1	25	1	AZT; PDN≤7.5mg /day; colchicine	AZT; PDN≤7.5mg /day; colchicine	No	39+5	Caesarean	3195	No	Yes (oral ulcers; fatigue)	No
**11**	Behçet's Disease	Mucocutaneous: oral and vaginal ulcers. Articular: arthralgias.	1	28	13	PDN≤7.5mg /day	PDN≤7.5mg /day	No	39+6	Eutocic	3650	No	No	Yes (arthralgias)
			2	30	15	PDN≤7.5mg /day	PDN≤7.5mg /day; colchicine	No	37+5	Dystocic	2805/3030 (twin gestation)*	No	No	No
**12**	Behçet's Disease	Mucocutaneous: oral and vaginal ulcers. Articular: arthralgias. Ophthalmologic: recurrent uveitis. Neurologic: CNS vasculitis.	1	34	5	AZT	AZT; colchicine	No	38	Caesarean	3580	No	Yes (oral ulcers)	No
			2	38	9	AZT	AZT	No	38+6	Caesarean	3616	No	No	No
**13**	Behçet's Disease	Mucocutaneous: oral and vaginal ulcers. Articular: arthralgias.	1	34	26	None	None	No	37+5	Caesarean	3155	No	No	No
**14**	Behçet's Disease	Mucocutaneous: oral and vaginal ulcers. Articular: arthralgias.	1	27	0	None	None	No	41	Caesarean	4010	No	No	No

**15**	Takayasu Arteritis	Vascular: thoracic aorta, supra-aortic vessels, bilateral carotids. Neurologic: stroke with hemiparesis. Ophthalmologic: retinal vasculitis. Cardiac: moderate aortic regurgitation.	1	21	3	Tocilizumab ;PDN>7.5mg /day	Tocilizumab ;PDN>7.5mg /day; AAS	No	37	Caesarean	2720	No	No	No
**16**	Takayasu Arteritis	Vascular: aortic arch, descending thoracic aorta, left common carotid.	1	34	4	AZT	AZT; PDN≤7.5mg /day; AAS	No	39+1	Dystocic	3160	No	No	No
			2	39	9	AZT; PDN≤7.5mg /day	AZT; PDN≤7.5mg /day; AAS	Yes (miscarriage at 7 WG)	*N/A*	*N/A*	*N/A*	No	No	No
**17**	Takayasu Arteritis	Vascular: full aortic axis and supra-aortic branches. Extra-vascular: granulomatous lymphadenitis, palmoplantar pustulosis.	1	20	0	None	PDN>7.5mg /day; AAS	No	38+5	Dystocic	2790	No	No	No
**18**	Takayasu Arteritis	Vascular: thoracic aortic aneurysm. Cardiac: severe aortic valve regurgitation.	1	23	5	Tocilizumab ; PDN>7.5mg /day	Tocilizumab ; AZT; PDN>7.5mg /day; AAS	Yes (preterm birth)	32+6	Caesarean	1900	No	Yes (fatigue; palpitations; orthostatic lypotimia)	No
**19**	Polyarteritis nodosa	Cutaneous: biopsy-confirmed lesions. Muscular: myalgias. Neurologic: paresthesias.	1	30	8	None	PDN≤7.5mg /day	Yes (FGR)	38+6	Eutocic	2810	No	Yes (fatigue, myalgias)	No
			2	32	10	None	PDN>7.5mg /day	No	37	Eutocic	3085	No	Yes (fatigue, myalgias, paresthesias)	No
**20**	Polyarteritis nodosa	Cutaneous: digital ulcers. Neurologic: mononeuropathy. Articular: arthritis.	1	36	1	AZT	HCQ; AZT; PDN>7.5mg /day; AAS	No	37+2	Caesarean	2755	Yes (fatigue)	No	No
**21**	Polyarteritis nodosa	Vascular: ophthalmic artery, celiac, mesenteric, renal arteries. Articular:	1	31	16	HCQ; AZT; PDN≤7.5mg /day	HCQ; AZT; PDN≤7.5mg /day; AAS	Yes (SGA, pre-eclampsia)	37+3	Eutocic	2440	No	No	No

		polyarthritis. Cutaneous: PAN-consistent biopsy.												
**22**	Polyarteritis nodosa	Cutaneous: livedo with biopsy-confirmed vasculitis. Vascular: Raynaud phenomenon and digital ulcers. Gastrointestinal: diarrhea. Neurologic: paresthesias.	1	29	0	None	HCQ; AZT; PDN≤7.5mg /day; AAS	No	37+5	Dystocic	2825	Yes (initial presentation during conception)	Yes (toe flexion paresis and digital ulcer)	No
**23**	Cryoglobulinaemic vasculitis	Constitutional: fever. Cutaneous: rash, livedo. Articular: arthritis.	1	32	13	None	PDN≤7.5mg /day	No	37+5	Eutocic	3020	No	No	No
			2	36	17	None	None	No	39	Eutocic	3020	No	No	No
**24**	Cryoglobulinaemic vasculitis associated with Sjögren’s syndrome	Cutaneous: vasculitic lesions. Glandular: recurrent parotiditis; xerostomia, xerophtalmia.	1	34	10	HCQ	HCQ	No	39	Caesarean	3000	No	No	No
**25**	ANCA-PR3 cutaneous vasculitis	Cutaneous: livedo with biopsy-confirmed vasculitis. Articular: arthralgias. Glandular: xerophtalmia.	1	20	5	HCQ; PDN>7.5mg /day	HCQ; PDN>7.5mg /day	Yes (SGA)	40	Eutocic	2845	No	Yes (livedo reticularis)	No
**26**	Relapsing Polychondritis	Cartilaginous: auricular and nasal chondritis.	1	33	1	PDN≤7.5mg /day	PDN≤7.5mg /day	Yes (SGA, FGR, preterm birth)	36	Caesarean	2040	No	Yes (auricular chondritis)	Yes (auricular chondritis)
**27**	IgA vasculitis	Cutaneous: purpura. Gastrointestinal: abdominal pain. Articular: arthralgias. Biopsy: leukocytoclastic vasculitis with IgA.	1	25	0	PDN≤7.5mg /day	PDN≤7.5mg /day	No	40+2	Eutocic	3435	No	No	No

**28**	Eosino-philic granuloma-tosis with polyangiitis	Respiratory: adult-onset asthma. Sinusitis. Articular: arthralgias. Hematologic: eosinophilia. Cutaneous: urticarial lesions.	1	40	5	PDN≤7.5mg /day	PDN≤7.5mg /day; AAS	Yes (miscarriage at 6 WG)	*N/A*	*N/A*	*N/A*	No	No	*N/A*

Abbreviations: APO, Adverse Pregnancy Outcomes; PDN, Prednisolone; AZT, Azathioprine; HCQ, Hydroxychloroquine; AAS, Acetylsalicylic Acid; WG, Weeks of Gestation; SGA, Small for Gestational Age; FGR, Fetal Growth Restriction; N/A, Not Applicable; MRI, Magnetic Resonance Imaging; CNS, Central Nervous System. * Twin pregnancy; both weights listed.

For Review Only

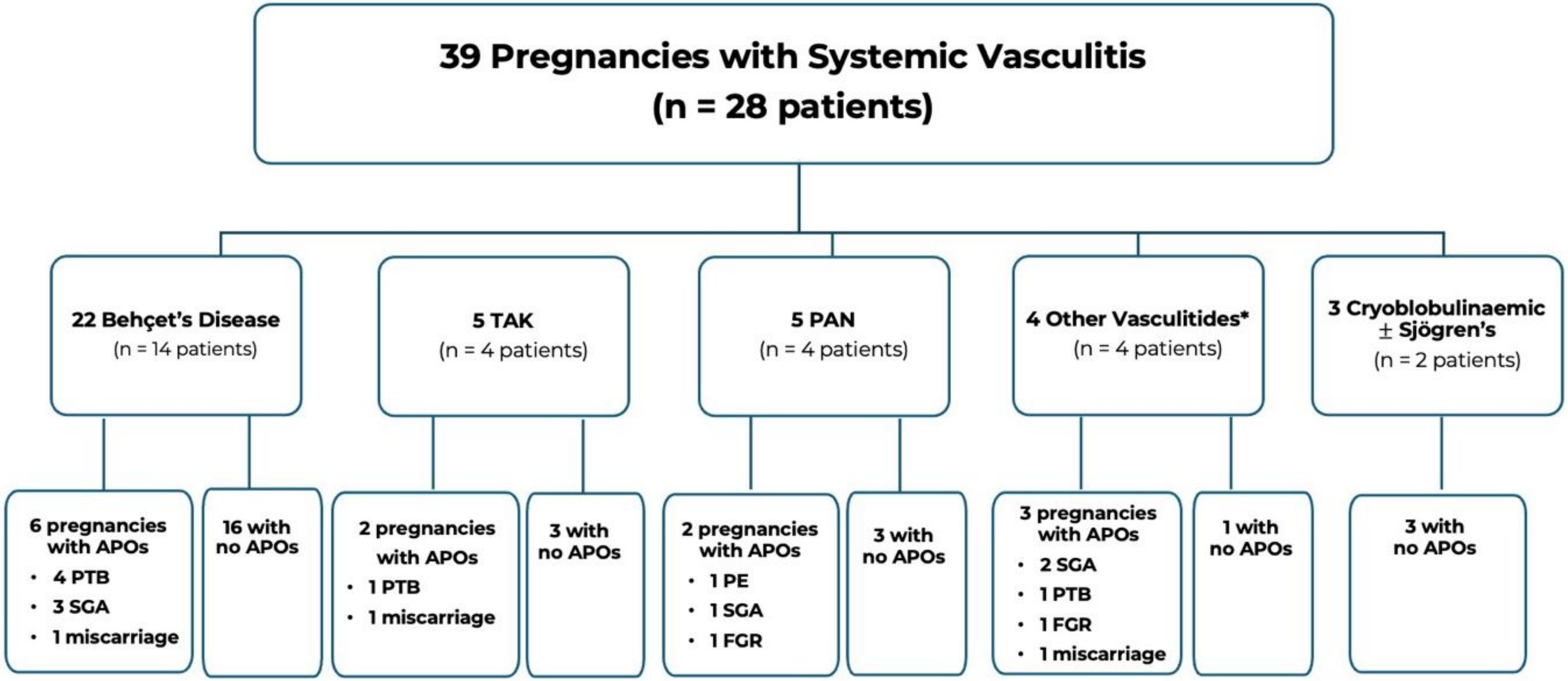

Supplementary Figure S1. Flowchart depicting the 39 pregnancies included in the study, distributed across the main subtypes of systemic vasculitis. For each subgroup, the total number of pregnancies and women is shown, together with the presence and type of adverse pregnancy outcomes (APOs), when applicable. For detailed APO combinations see Supplementary Table S1.
*Other vasculitides include IgA vasculitis, ANCA-PR3 cutaneous vasculitis, relapsing polychondritis, and eosinophilic granulomatosis with polyangiitis.

Abbreviations: APOs, adverse pregnancy outcomes; PAN, polyarteritis nodosa; TAK, Takayasu arteritis; FGR, fetal growth restriction; SGA, small for gestational age; PTB, preterm birth; PE, pre-eclampsia.
